# Cerebral Tumor Involving the Nerves of the Special Senses

**Published:** 1882-02

**Authors:** Lucien Howe


### Clinical Reports.

### Cerebral Tumor involving the Nerves of the Special Senses.

By Lucien Howe, M. D.

On the 21st of August, 1880, I was consulted by A—— M——, a man 27 years old, on account of the imperfect condition of his vision. For a year previous he had been troubled with headaches, always located in the frontal region, and with the hope of finding relief from them, he had applied to several physicians. Among others he consulted Dr. Mynter, who, observing the faulty vision, referred him to me.

Externally, there was nothing abnormal about the eyes, except that the pupils were rather large; but a glance with the ophthalmoscope at the retina and optic nerve showed these to be much inflamed. In the fundus of the right the abnormal changes were especially well marked, the outline of the optic disk being ill-defined, the vessels enlarged and tortuous, and when traced from their starting points, were often lost from sight, covered at intervals by the œdematous and swollen retinal tissue. With this eye, however, the patient could still count fingers five feet distant and could read No. 14 of Jaeger's test types.

The interior of the other eye presented a similar appearance, except that the nerve had not become involved to so great an extent. With this, the vision for the distance was still one-fifth of the normal, and for near objects was no worse than its fellow. The general health of the patient seemed to be quite good. In early youth he had suffered from disease of the hip-joint, and the resulting lameness had led to sedentary habits, so that his muscles were rather flabby and the skin pale; but he had an excellent appetite, slept well, could hear perfectly, and but for the headaches and imperfect vision, would have had few complaints to make. He denied ever having had syphilis, and there were no outward appearances to indicate it. A very unfavorable prognosis was given, and the patient, becoming alarmed, disappeared temporarily from under my observation, and partly neglected the few therapeutic measures which were advised. Subsequently he returned, and an effort was made by means of complete protection from the light by counter-irritation, depletion and alteratives, to lessen the inflammatory process going on in the retina and optic nerve, with the hope that even if the cause of the trouble could not be reached, at least that its effect might not be so injurious.

Such expectations, however, proved groundless, and as the vision grew gradually worse, it was evident that no improvement could ever be obtained, and that the case was interesting only from a pathological standpoint.

On the 14th day after his first visit, the vision of the right had been reduced to mere perception of light, and later was lost entirely. On the 33d day the vision of left had sunk to less than one-twentieth of the normal; on the 37th day he could only count fingers at four feet, and before the end of the third month this eye, like its fellow, was totally blind. At the same time the hearing began to be affected. It was difficult to determine on which side this commenced, but the morbid process involved the auditory nerves more rapidly than the optics, and before the end of the sixth month he was also completely deaf. The sense of smell was likewise impaired, and Dr. Mynter informs me that he has held beneath the patient's nostril a vial of aqua ammonia without any sign being given to indicate that it was at all disagreeable or irritating.

Finally the taste evidently was lost, one article of food being relished quite as much as another. There remained, of course, only the sense of touch with which to communicate with those about him. In this miserable condition the patient lingered for nearly nine months, gradually becoming incoherent in his speech, growing more emaciated and losing strength, till at last he died, on the 28th of December.

A post-mortem examination was made by Dr. Mynter and others thirty-six hours afterwards. The calvaria was found to be remarkably thin, and the lining membrane denuded in various portions. In attempting to dislodge the front part of the brain it was found to be adherent to the underlying dura mater, especially on the right side, and in the vicinity of the cribriform plate of the ethmoid. Further inspection revealed in this locality a well-defined tumor, involving the inferior portion of the right anterior lobe. This tumor was about the size and shape of a large egg, the longer diameter lying in an antero-posterior direction and the side of the tumor extending to the longitudinal fissure. It was of rather a dark-red color as compared with the neighboring brain tissue, and firm and unresisting upon pressure. There was naturally considerable hyperæmia in that vicinity, but the vasularity could not, with the naked eye, be seen to extend further back than the anterior part of the pons. A microscopical examination of the tumor showed its structure to be that of a fibro-sarcoma. The point of interest in connection with this case is the extent to which the nerves of the special senses were affected, while others in the immediate vicinity were apparently healthy. From the location of the tumor it is not surprising that the sense of smell was lost, and if care had been taken in this part of the examination, probably some defect would have been found at an earlier stage. It was also natural that the optic nerves should have become involved in the order, and to the great degree noticed.

But it will be remembered that the portio mollis emerges from the brain substance posteriorily to the third, fourth and fifth nerves, externally to the sixth, and in very close proximity to the portio dura and glosso-pharyngeal, and then passes onwards to its destination in the labyrinth. It is therefore difficult to understand how this nerve on both sides could have its function entirely destroyed, while those just mentioned, viz, the motor oculi, patheticus and trigeminus, the abducens, the portio dura and the glosso-pharyngeal, were apparently in perfect condition.

Not the least sign of paralysis about the muscles of the eye, nor elsewhere, nor any diminished acuteness of sensation could be perceived during life, and after death these nerves appeared also to be in a healthy condition. It is, of course, not supposed that the tumor itself had any specific action in producing these effects upon the nerves of special sense, but it is strange that these, and particularly the auditory nerves should be thus singled out among their neighbors; and I am not aware of a similar condition having been before noticed.

